# Supplementary material for: Genotype-matched Newcastle disease virus vaccine confers improved protection against genotype XII challenge: The importance of cytoplasmic tails in viral replication and vaccine design
Source: PLoS One. 2019 Nov 14;14(11):e0209539. doi: 10.1371/journal.pone.0209539 (PMC6855454; doi:10.1371/journal.pone.0209539)
Supplement: S1 Table — (DOCX) [file pone.0209539.s001.docx]

| Group | Number of viral shedding chickens (positive/total) | | | | | |
| --- | --- | --- | --- | --- | --- | --- |
|  | 2 d.p.c.^(a)^ | | 4 d.p.c. | | 7 d.p.c. | |
|  | Oral | Cloacal | Oral | Cloacal | Oral | Cloacal |
| rLS1 | 0/14 | 1/14 | 1/14 | 3/14 | 0/14 | 0/14 |
| rLS1-XII-1 | 7/14 | 10/14 | 9/13 | 9/13 | 4/10 | 7/10 |
| rLS1-XII-2 | 0/14 | 0/14 | 0/14 | 1/14 | 0/14 | 0/14 |
| PBS | 9/10 | 10/10 | 5/5 | 5/5 | NS^(b)^ | NS |

1. d.p.c = days post challenge
2. NS = no survivors
